# Does implementation of an early warning trigger system result in earlier administration of antibiotics for septic patients?

**DOI:** 10.1186/1757-7241-20-S2-O3

**Published:** 2012-04-16

**Authors:** Julie Mackenhauer, Anne-Katrine Bertelsen, Helle Lykkeskov Nibro, Troels Krarup Hansen

**Affiliations:** 1 Research Center for Emergency Medicine, Aarhus University Hospital, Denmark; 2 Intensive Care Unit, Noerrebrogade, Aarhus University Hospital, Denmark

## Background

Early treatment of sepsis has lead to improved outcomes, making early identification of the condition essential. The early warning trigger system TOKS (Tidlig Opsporing af Kritisk Sygdom), including systematically daily recordings of vital signs, was implemented November 1st 2009 throughout Aarhus University Hospital. We hypothesized that the use of TOKS would result in earlier administration of antibiotics for septic patients. The aim of the present study was to compare the degree of appropriately initiated therapy for septic patients admitted to the ICU before and after introduction of TOKS.

## Methods

Our cohort of septic patients was identified through a prospective screening of all patients admitted to the ICU (a 12-bed, tertiary intensive care unit receiving approx. 900 patients/year) from the Emergency Department (ED) and wards. We included all septic patients admitted 3 and 10 months before and after TOKS, respectively. We reviewed patient charts regarding timing of appropriate sepsis therapy (antibiotic administration and blood cultures).

## Results

We identified 78 patients with sepsis admitted to the ICU (46 in the before- and 32 in the after TOKS groups), accounting for 25.3% resp. 18.1% of all ICU admissions (p=0.09). A total of 49 patients (63%) were admitted directly from the ED. There were no differences regarding age and gender between the two groups. Overall in-hospital mortality was 24.4%.

Appropriate antibiotic treatment was initiated prior to ICU-admission in 37% of patients before and 66% after implementation of TOKS (p=0.02). Blood cultures were obtained prior to ICU-admission in 51% resp. 67% of cases (p=0.22). Compared to current hospital guidelines, antibiotics were delayed in 25% of cases before TOKS compared to 12% after (p=0.21).

## Conclusion

We found that significantly more patients received appropriate antibiotic treatment prior to admission to the ICU after implementation of an early warning trigger system in the ED and wards. With the increasing focus on sepsis treatment, it is not possible to rule out a time effect, but our observational data may indicate that the use of TOKS leads to earlier administration of antibiotic treatment in patients with sepsis.

